# The Role of Lung Ultrasonography in Etiologic Diagnosis of Acute Dyspnea in a Resource Limited Setting

**DOI:** 10.30476/BEAT.2020.46453

**Published:** 2020-04

**Authors:** Nguyen The Nguyen Phung, Trang Thi Thanh Vo, Kam Lun Ellis Hon

**Affiliations:** 1 *Department of Pediatrics, University of Medicine and Pharmacy at Ho Chi Minh city, Viet Nam.*; 2 *Children Hospital 1 in Ho Chi Minh city, Viet Nam.*; 3 *The Hong Kong Children's Hospital, Hong Kong*

**Keywords:** Acute dyspnea, Lung ultrasound, Pneumonia, Pleural effusion, Pneumothorax, Acute pulmonary edema

## Abstract

The aim of the current study was to describe lung ultrasonography (LUS) characteristics and to evaluate the agreement between LUS and chest radiography (CXR) in diagnosis of four conditions causing most acute dyspnea in children, namely, pneumonia, pleural effusion, pneumothorax and acute pulmonary edema in children at a teaching hospital in Vietnam. We reviewed the records of the chidren between January and June 2018, who presented to emergency department (ED) or pediatric intensive care unit (PICU) at children hospital 1 (CH1) with acute dyspnea and had final diagnosis of one of four etiologies including pneumonia, pleural effusion, pneumothorax and acute pulmonary edema. All patients underwent CXR and LUS at the time of admission. Eighty-one children with acute dyspnea including pneumonia (n=65, 80%), pleural effusion (n=9, 11%), pneumothorax (n=3, 4%) and acute pulmonary edema (n=4, 5%) were enrolled. LUS was identified among 100% of cases with pleural effusion and pneumothorax (CXR only showed 73.3% and 50%, respectively); 92.3% of cases with pneumonia (CXR showed 93.8%) and only 75% of cases with acute pulmonary edema (CXR showed 50%). When comparing LUS with CXR, we noticed a good agreeement between the 2 methods in the diagnosis of pneumonia (kappa=0.64, *p*<0.001). LUS was shown to be a feasible and non-invasive technique which can help clinicians to comfirm the etiology of acute pulmonary dyspnea.

Acute dyspnea is a common clinical presentations in the emergency department (ED) [1]. Four etiologies of acute dyspnea in children were pneumonia, pleural effusion, pneumothorax and acute pulmonary edema. In the ED setting, an estimated of 40% of patients underwent CXR [2-4]. CXR provides diagnostic sensitivity of about 60%; and associated with a risk of radiation exposure in children and pregnant women [5]. Chest *computed tomography* (CT*) scan *plays a vital role in the diagnosis of various lung diseases, but is less readily accessible in many developing countries. It may be the source of 0.4% of all cancers in the general population due to risk of radiation exposure [6,7]. Lung Ultrasonograpy (LUS) is a point-of-care application which is more sensitive and specific than CXR, and has the advantage of being a fast and non-radiating technique [1,7-9]. So this study aimed to evaluate the agreement between LUS and CXR in the diagnosis of conditions causing acute dyspnea at Children Hospital 1(CH1). 

From January to June 2018, children presented to ED with acute dyspnea that required oxygen therapy were admitted. These patients had initial diagnosis based on clinical features, laboratory tests, CXR or chest CT scan. At first evaluation, patients underwent LUS by pediatricians at ED. Final diagnosis of four conditions was based clinically on decision of pulmonary experts which served as gold standard criteria for this research. Inclusion criteria included age (2 months-15 years), dyspnea/progressive dyspnea, and informed written consent from parents. Congenital cardiac diseases and chronic pulmonary diseases were excluded. The CXR and chest CT scans were evaluated by the expert pediatric radiologist. LUS was conducted immediately after CXR. Eighty-one patients were recruited: 65 cases (80%) of pneumonia, 9 cases (11%) of pleural effusion, 3 cases (4%) of pneumothorax and 4 cases (5%) of acute pulmonary edema. Among patients with pneumonia, 6 cases were complicated with pleural effusion and 1 case with pneumothorax.

Totally, 51.8% of admitted patients were with chief complaint of dyspnea. In initial assessment, 53.1% of children were classified as servere dyspnea, and respiratory support and 42% were on mechanical ventilation and 23.5% were on nasal continuous positive airway pressure. Overal mortality rate was 25%. Among patients with pneumonia, distribution of specific features was cough (90.8%), fever (75.4%) and crackles (86.1%). 43% of children had premorbid diseases including cerebral palsy (39.2%), anemia (36.9%), Down syndrome (10.7%), leukemias (7.1%) and miscellaneous diseases (6.1%). LUS revealed pulmonary abnormalities consistent with pneumonia in 60/65 children (92.3%). Findings of LUS with pneumonia (n=65) were shown in Table 1. Totally, 90.8% of children showed the same positive pneumonia on both LUS and CXR. We noticed a good agreeement between the two methods in the diagnosis of pneumonia (Table 2, kappa=0.64; *p*<0.001). High rate of specific findings of LUS were seen in diagnosis with pleural effusion, these were quad sign (93.3%), sinusoid sign (73.3%), mirror sign and spine sign (86.7%). The stratosphere signs and lack of lung sliding were observed in all of cases with pneumothorax, and lung point only occurred in 50% patients. LUS identified 100% of cases with pleural effusion and pneumothorax, CXR only showed 73.3% and 50%, respectively. LUS identified 75% of cases with acute pulmonary edema, while CXR showed 50%. LUS and CXR showed the same positive diagnosis for 73.3%, 50% and 50% cases with pleural effusion, pneumothorax and pulmonary edema; respectively.

The diagnosis of pneumonia in children is historically based on physical examinations. CXR may not be performed routinely in ambulatory cases [10, 11]. In our study, only lung consolidation (63%) was recognized by LUS as marked liver-like appearance with an irregular lower boundary and with arborized hyperechogenic areas within**.** The rate of lung consolidation with dynamic air bronchogram in our study was greater than previous reports [10]. Chen *et al*. [11] showed pneumonia on LUS with consolidation whose size was greater than 1 cm, but not other standard criteria (subpleural consolidations and B lines). Geurra *et al*. demonstrated the findings of LUS for pneumonia including both lung consolidation (size>1 cm) and subpleural consolidations (<1 cm). So positive pneumonia on LUS was the same as our study. Sensitivity and specificity of the sign “lung consolidation” were high in the diagnosis of pneumonia [9, 12].

Claes *et al*. [13] compared consolidation size on LUS with CXR and reported the areas of consolidation identified only on LUS to be significantly smaller than on CXR. Urbankowski *et al.* illustrated a strong correlation between the dimensions of the consolidation in three axes, suggesting that one-dimension measurement could represent overall consolidation size [14]. Our study revealed that 91% (59/65) of cases showed a positive pneumonia on both LUS and CXR, and demonstrated a good agreement in diagnosis of pneumonia between two methods.

Regarding LUS findings for pleural effusion, only 73.3% of cases revealed sinusois sign due to above large effusion and fibrinosis. There were 2 false negative spine sign and mirror sign because of fibrotic pleural effusion and focal effusion. Agreement between LUS and CXR of pleural effusion based on final diagnosis was good [15]. Regarding LUS findings for pneumothorax in our study, 100% of cases with pneumothorax had lack of lung sliding. Daniel Lichtenstein demonstrated that 95.1% of patients with absence of lung sliding had high sensitivity and specificity of 95.3% and 91.1%, respectively, and negative predictive value (NPV) was 100% (*p*<0.001) in diagnosis of pneumothorax [16]. 

Wilkerson *et al.* showed if diagnosis of pneumothorax was only based on being lack of lung sliding, posistive predictive value (PPV) would be from 87% to 27%. Sensitivity and specificity increased to 100% and 95%; respectively and PPV and NPV increased to 85% and 100%, respectively when “no lung sliding” sign was combined with no B lines [17]. Ultrasonographic findings was similar to chest CT scan in determining pneumothorax and estimating the volume of gas in pleural space (kappa=0.67;* p*<0.001) [18]. 

LUS showed high sensitivity, specificity, PPV and NPV (90-98%); whereas CXR had sensitivity and specificity of 50.2% and 99.4%, respectively and PPV and NPV ​​of 96.5% and 85.6 %, respectively [19]. Considering LUS findings for acute pulmonary edema, CXR can aid in identifying pulmonary edema, but the overall accuracy of the presence or absence of congestive heart failure on radiographs may be as low as 69% [20]. LUS revealed 75% of children with acute pulmonary edema in our study. A negative point-of-care LUS can almost exclude acute pulmonary edema [8]. 

One study showed a good agreement of two methods for acute pulmonary edema (kappa=0.59;* p*<0.001) [20]. A structured LUS examination can detect common pulmonary diseases causing acute dyspnea in children with the similar accuracy and reliability as CXR. LUS may fill an important diagnostic gap for children presenting with suspected pneumonia.

**Table 1:** Findings of LUS by final diagnosis of pneumonia (N=65).

**Findings**	**Frequency**
Pleural line abnormalities (thickening, irregularity, hypoechogenicity)	13 (20%)
Lack of lung sliding	4 (6.2%)
B line	61 (93.8%)
One side	54 (88.5%)
Both sides	7 (11.5%)
Number of B line	
< 3 B lines	23 (35.4%)
≥ 3 B lines	38 (58.5%)
Lung consolidation (>1cm)	41 (63.1%)
Dynamic air bronchogram	36 (55.4%)
Fluid bronchogram	5 (7.7%)
Subpleural consolidations (< 1cm)	26 (40%)
Morphology of sonographic patterns	
B lines on both sides+lack of lung sliding	4 (6.1%)
B lines on one or both sides+subpleural consolidations±pleural line abnormalities.	20 (30.7%)
Lung consolidation with air bronchograms±B lines	36 (55.4%)
Normal pattern (none of above)	5 (7.7%)

***(*** *Note: number of B lines≥3)*

**Table 2:** Agreement between CXR and LUS by final diagnosis of pneumonia

**Pneumonia (N=65)**		**LUS**		**Total**
		**Positive (+)**	**Negative (-)**	
CXR	Positive (+)	59	2	61
	Negative (-)	1	3	4
Total		60	5	65

## Conflict of Interest:

 None declared. 
